# High intragenomic, intergenomic, and phenotypic diversity in pulcherrimin-producing *Metschnikowia* yeasts indicates a special mode of genome evolution

**DOI:** 10.1038/s41598-024-61335-5

**Published:** 2024-05-08

**Authors:** Matthias Sipiczki, Kinga Czentye, Zoltán Kállai

**Affiliations:** 1 Department of Genetics and Applied Microbiology, University of Debrecen, Debrecen, Hungary; 2 Present Address: Institute of Horticulture, University of Debrecen, Debrecen, Hungary

**Keywords:** Barcode diversity, Chimeric genome, Antifungal antagonism, Pulcherrimin, Dimorphic transitions, Invasive growth, Biotechnology, Evolution, Genetics, Microbiology

## Abstract

In molecular systematics, the delimitation of yeast species is based on the notion that the barcode differences are smaller within species than between them. The most widely used barcodes are segments of the chromosomal repeats coding for ribosomal RNAs that are homogenised in yeasts. The analysis of these segments of the type strains of ten species recently merged in *Metschnikowia pulcherrima* and 37 new isolates demonstrated that this is not the case in this species. The intragenomic diversity significantly exceeded the threshold gaps used to differentiate related yeast species. Large segments of the D1/D2 domains were not diverse within the genomes and could therefore be used to determine the taxonomic affiliation of the isolates. The genome structures of the isolates were compared by RAPD and the RFLP of the mitochondrial DNA. Both patterns were highly heterogeneous. The sequence analysis of the *PUL4* gene (a member of the *PUL* gene cluster involved in pulcherrimin production) revealed very high intragenomic differences, suggesting that the genomes may be chimerised. Three phenotypic traits related to the antimicrobial antagonism characteristic of the species were also highly diverse and prone to reversible segregation resembling epigenetic processes (silencing and reactivation of regulators) rather than mutations and back-mutations. These features make *M. pulcherrima* unique among yeasts and indicate that it evolves in a non-standard way.

## Introduction

Species delimitation in yeasts is based on the combination of barcode DNA sequence analysis and the examination of selected physiological, biological and morphological properties^1^. The most important barcodes are segments of the chromosomal repeats that code for rRNA (primary barcodes) and segments of genes of certain conserved proteins (secondary barcodes). The barcode sequences are identical or highly similar in conspecific organisms and different in allospecific organisms^2,3^. The latter difference is the barcode gap, on which the taxonomic differentiation of species is based.

The application of the barcodes is based on the assumptions that (1) the rRNA cistrons (repeats) containing the primary barcodes are homogenised across the repeat array(s) in the genome of an organism to ensure that all have identical nucleotide sequences^4^ and (2) the secondary barcodes are present in single copies or in more but identical copies (no heterozygosity)^1^.

Recent analyses of the barcodes of the type strains of ten pulcherrimin-producing *Metschnikowia* species (grouped in the *pulcherrima* clade) found no clear barcode gaps between them due to very high intra-genomic (intra-strain) diversity of the sequences^5,6^. The intra-genomic sequence diversity is reflected in high numbers of “ambiguous nucleotides” (dimorphic, rarely polymorphic sites) in amplicon sequencing. Individual barcode sequences cloned from the amplified barcode DNA of a strain often differed much more from each other than from sequences cloned from strains of other type strains of the clade. This phenomenon is rather unusual in yeasts and renders the determination of the taxonomic affiliation of strains and their differentiation from each other practically impossible. Because of the lack of clear barcode gaps the ten species of the clade were merged in one species under the taxonomic name *M. pulcherrima*^6^. However, the merged species is highly heterogeneous due the extremely high inter- and intragenomic primary barcode diversity. The degree of diversity is exceptionally high compared to other yeast species and was hypothesised to be attributable to a birth-and-death mechanism of repeat evolution instead of array homogenisation^7^. The analysis of the secondary barcodes and additional 28 protein-encoding genes of four strains for which complete genome sequences were available revealed heterozygosity for all of them. The analysed genes had at least two copies in the genomes which differed in sequence and phylogenetic history. These copies often differed from each other more than from their counterparts in other strains (higher intra-genomic than inter-genomic differences). Certain genes containing secondary barcode segments were found in more than two copies, all differing in sequence, indicating aneuploidy. From these results it was concluded that the genomes of the type strains of the merged species are chimeras composed of variable copies of genes of different phylogenetic histories^5,6^.

*M. pulcherrima* gained attention due to the antimicrobial activity of its strains which can be exploited in protecting crops from harmful (e.g. rotting) microorganisms (for a review, see^8^). Numerous yeast species are known to exert adverse effects on other microorganisms but *M pulcherrima* stands out with the extent and strength of its antimicrobial antagonism. Several antagonistic mechanisms have already been revealed and investigated in yeasts (for recent reviews, see^9,10^. One mechanism is related to the production of pulcherrimin, a red complex, a characteristic feature of *M. pulcherrima* and many *Kluyveromyces* strains [e.g.^11–13^]. Pulcherrimin is a complex of pulcherriminic acid and ferric ions^14^. Pulcherriminic acid is a hydroxamic acid derivative of the cyclic dipeptide cyclo(l-leucyl-l-leucyl)^15^. It harbours two hydroxamate groups and forms pulcherrimin upon binding Fe_3_^14,16,17^). The reaction can take place within the cytoplasm of the yeast cells and also extracellularly because the pulcherriminic acid can be secreted into the environment. Since pulcherrimin is a coloured substance, its accumulation in the cytoplasm turns the colony dark red, whereas its formation outside the cells produces a pigmented halo in the medium around the colony^16^.

The biosynthesis and secretion of pulcherriminic acid has been investigated in detail in *Bacillus subtilis* and *B. licheniformis* (for a review, see^18^) and a similar pathway is assumed to operate in *K. lactis* and *M. pulcherrima* (for a review, see^8^). In the genomes of the latter organisms, four genes organised in the group called the *PUL* cluster encode proteins participating in the production of pulcherriminic acid and its release in the environment^8,13^.

As the reaction of pulcherriminic acid with ferric ions is irreversible^19^ and pulcherrimin is insoluble in water, the process is unlikely to play a role in iron acquisition. The chelated iron in pulcherrimin is inaccessible to the biochemical processes of microorganisms. Since iron is required for the activity of many proteins and cellular processes, its immobilisation by pulcherriminic acid adversely affects the propagation of many microorganisms and can even be lethal to their cells^12^. Due to their antimicrobial activities, the pulcherrimin-producing *M. pulcherrima* strains can be used as biological agents to protect agricultural commodities and food products against pathogenic and destructive microorganisms (for a review, see^8^). The strength of their antagonistic effect correlates with the intensity of the extracellular production of pulcherrimin (e.g.^12,20–22^).

The aim of this study was to investigate how characteristic the high genomic and physiological diversity observed in the type strains of the merged species is for *M. pulcherrima*. For the investigation, 37 “wild” strains were isolated in 8 localities from various fruits which are natural habitats for this species. Their molecular examination revealed high intragenomic (intrastrain), intergenomic (interstrain) molecular diversity and diverse genome structures. All strains had diverse primary barcode sequences and diverse copies of the *PUL4* gene (one of the four genes of the *PUL* cluster) in their genomes. Almost all strains had unique RAPD patterns and significant differences were detected between their mitochondrial genomes. Three phenotypic traits relevant for bioprotection namely pulcherrimin production, antifungal antagonism, and substrate invasion were also diverse and prone to reversible segregation resembling epigenetic processes. However, no clear correlation was observed between the genomic and the phenotypic diversities. Apart from the revealed very high diversity, this study also finds that the *M. pulcherrima* strains can switch to invasive pseudohyphal growth. This property has been overlooked in previous studies, although it may significantly contribute to the biological protective power of *Metschnikowia* strains by sticking their pseudohyphae to injuries on the plant surface.

## Results

### Strain isolation

To isolate pulcherrimin-producing *Metschnikowia* strains, fruits were collected in the years 2022 and 2023. Supplementary Table S1 lists the fruit samples from which pigmented colonies were obtained. One colony was isolated for each fruit sample if the colonies were homogeneous in colour intensity on the FeCl_3_-containing plates and two colonies were isolated when the colour intensity was heterogeneous. Such pairs are marked with “a” and “b” in the table. 37 isolates were retained for the diversity analysis.

### Genetic-genomic diversity

#### The barcode sequences of the chromosomal repeats coding for rRNA are diverse both within the genomes and in comparison with other genomes (isolates)

**Figure 1:**
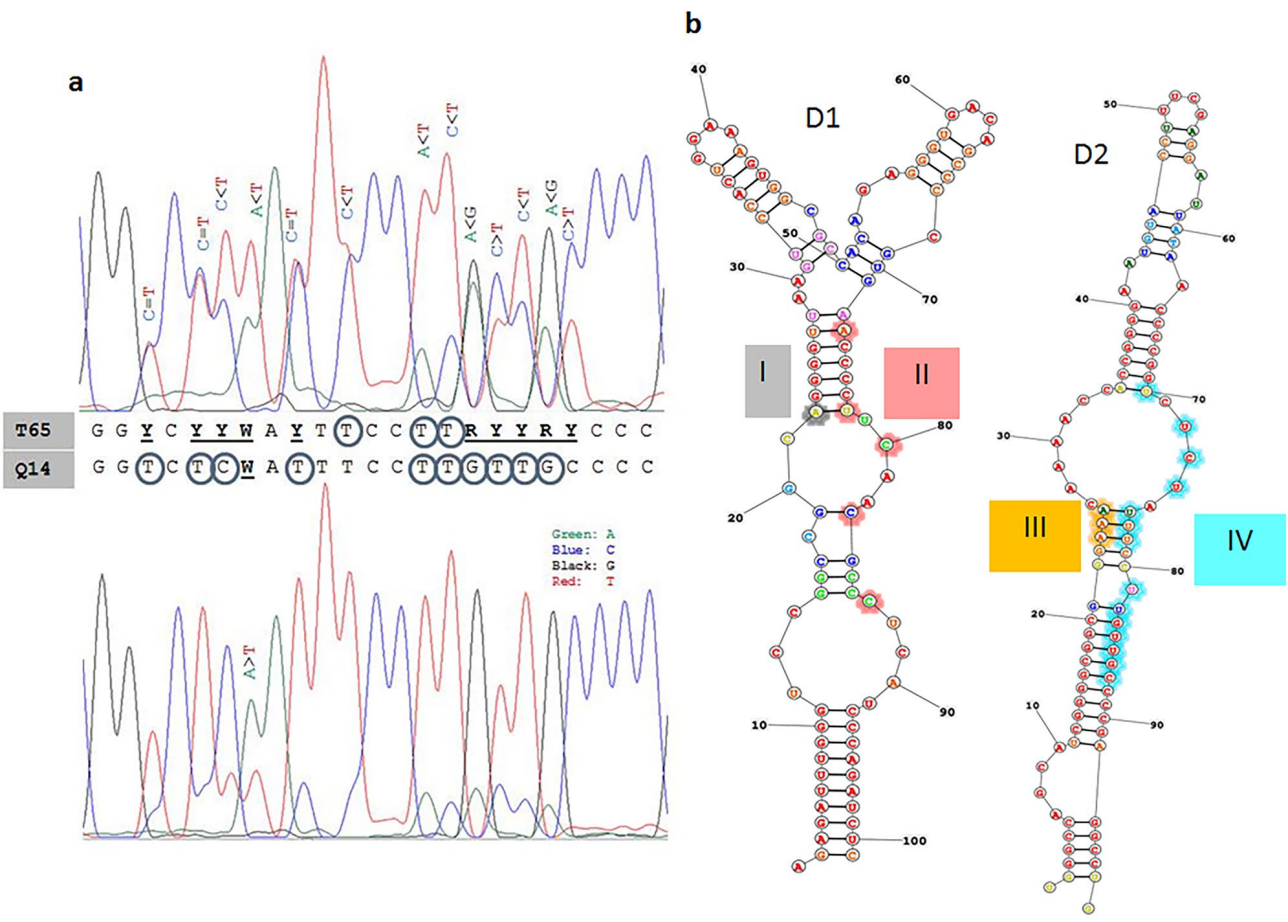
Intragenomic diversity of primary barcodes. (**a**) Double peaks in D1/D2 chromatograms. The variable blocks IV (see Table 1) of the isolates Z65 and Q14 are shown. Ambiguous nucleotides are underlined. Note that ambiguous (dimorphic) symbol is shown when both peaks are high. When one peak is much higher than the other, only the nucleotide that corresponds to the higher peak is shown. This nucleotide occurs more frequently but not exclusively in the amplified DNA molecules. The majority nucleotides are marked with circles. (**b)** Locations of the blocks of nucleotide variability in the predicted secondary structures of D1 and D2 hairpin-stem loops. The most variable positions (see Table 1) are marked with coloured background.

The D1/D2 domains of the genes coding for the large subunit rRNA and the ITS1-5S-ITS2 segments were amplified and sequenced with the Sanger method. All sequences contained ambiguous nucleotides at numerous positions and double peaks in even more positions of the chromatogrammes (Fig. 1a). In the latter cases, the peaks were different in height, and the nucleotides corresponding to the higher peaks were shown in the final sequence. All variable positions were SNDs (single nucleotide dimorphisms), mostly transitions Y (C/T) and R (A/G). No SNP (single nucleotide polymorphism) was detected.

In the D1/D2 sequences, the variable positions were concentrated in four blocks (I, II, III and IV in Table 1). The sequence of the block IV of an isolate with high number of ambiguous nucleotides and the sequence of the isolate with the lowest number of ambiguous nucleotides are compared in Fig. 1a. All blocks are located in hairpin-stem loops of the secondary structures of the transcripts (Fig. 1b).

**Table 1:**
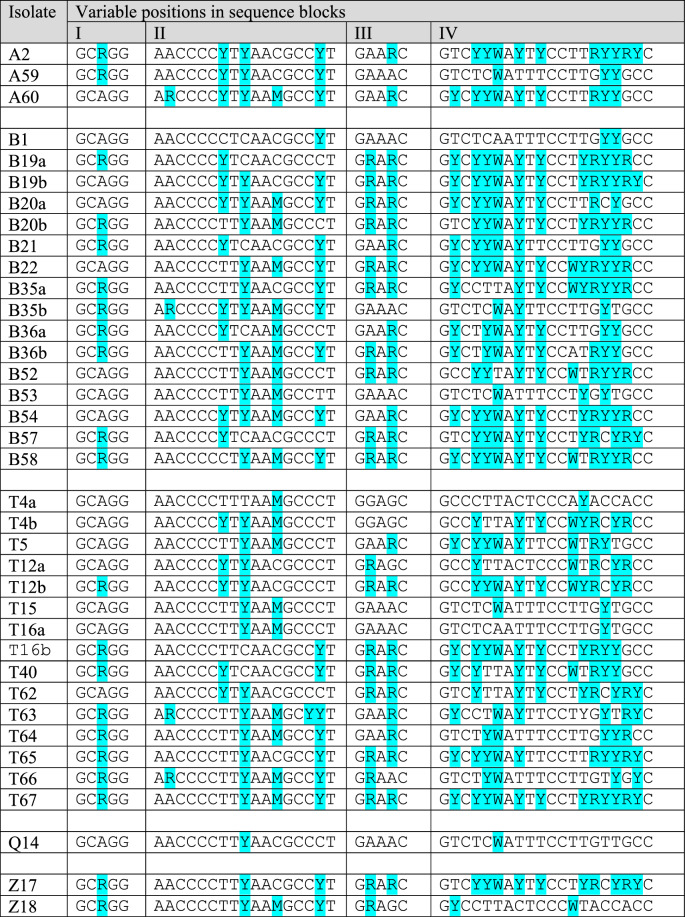
Intragenomic variability in D1/D2 barcode sequences.

Ambiguous nucleotides (SND sites) are marked with blue background. SND symbols: M: A or C (transversion), R: A or G (transition), Y: C or T (transition), W: A or T (transversion).

The intragenomic diversity of the ITS segments was much higher. Very few positions had identical nucleotides in all ITS1 sequences and indels occurred frequently in ITS2. In contrast to the dimorphism of the variable positions of the D1/D2 domains, three or four nucleotides alternated in certain positions of the ITS1 segments (SNT or SNP sites). The ITS2 segments contained much fewer ambiguous nucleotides but much more indels (Table 2). Because of their high intragenomic diversity, the ITS sequences were not deposited in databases.

**Table 2:**
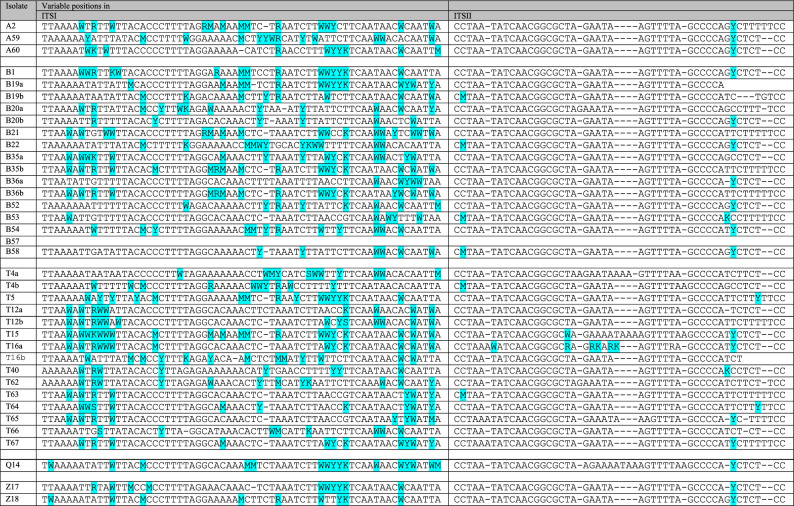
Intragenomic variability of ITS barcode sequences.

Ambiguous nucleotides are marked with blue background. SND symbols: SND symbols: K: G or T (transversion), M: A or C (transversion), R: A or G (transition), S: C or G (transversion), Y: C or T (transition), W: A or T (transversion).

Blast searches with the D1/D2 sequences in INSDC databases identified the sequences of *M. pulcherrima* CBS 5833^T^ (NRRL Y-7111^T^) as the most similar type-strain sequences. The identity varied between 93 and 99%, depending on the numbers of ambiguous positions in the sequence of the isolate and in the database sequence. Searches with ITS sequences lead to ambiguous results in certain isolates: low coverage, low percentage of identity with the *M. pulcherrima* type-strain sequences or higher identity with type strains of related species.

#### The genomes of most isolates have unique RAPD patterns

**Figure 2:**
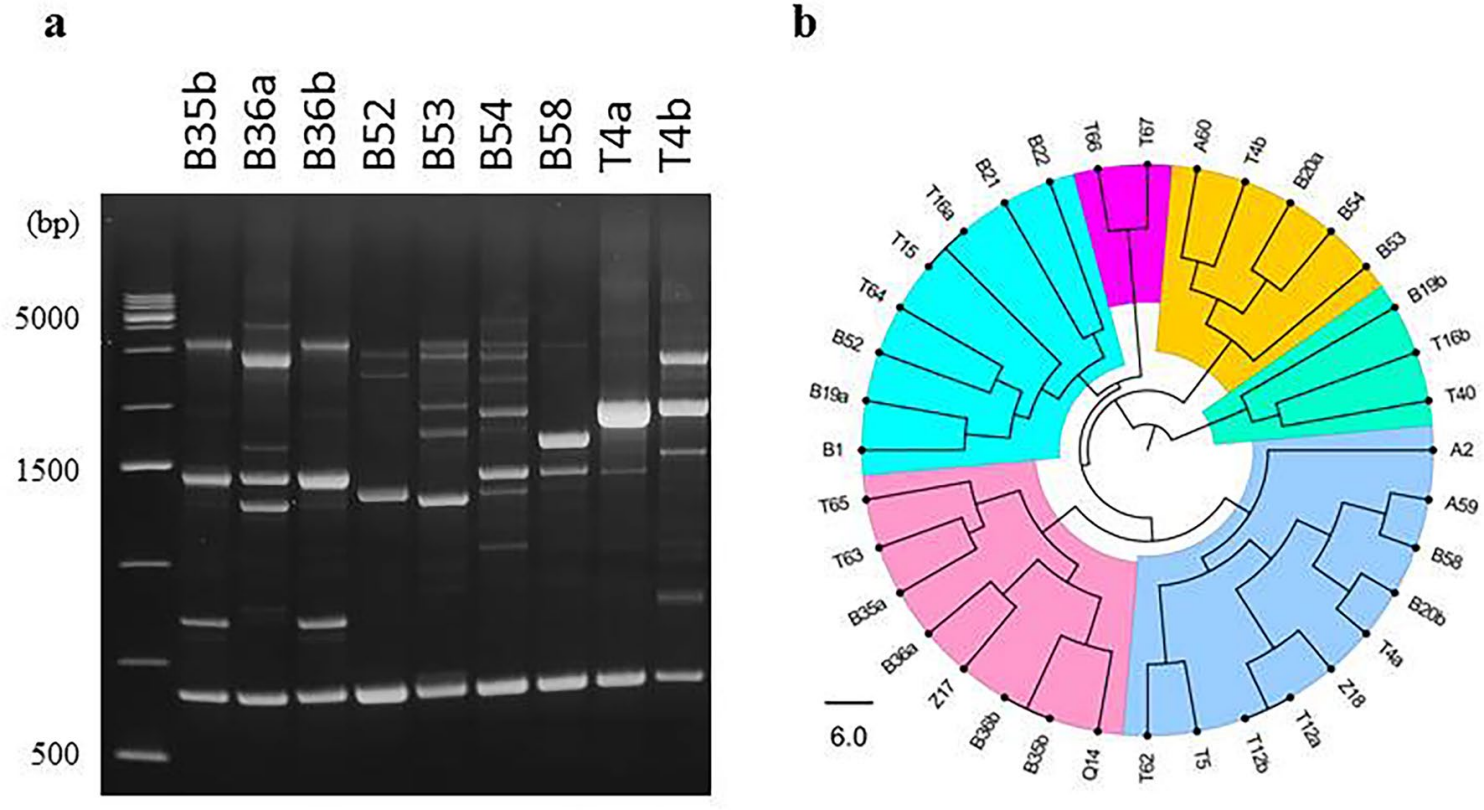
RAPD patterns of isolates. (**a**) Example band patterns. (**b**) UPGMA dendrogramme of the patterns of all isolates. The grouping of the isolates (coloured clades) does not correlate with their origin.

To compare the genomes of the isolates, PCR reactions were performed with two primers widely used for diversity studies. Both primers generated diverse banding patterns but the number of the fragments was higher when RAPD24 was used. Almost all strains had unique RAPD24 patterns. Figure 2 shows a dendrogramme inferred from the presence of 20 bands. All but three pairs of strains could be differentiated by comparing their banding patterns. One pair was isolated from the same sample suggesting that they might have been members of the same clone. Interestingly, their barcode sequences differed both in the D1/D2 (Table 1) and in the ITS segments (Table 2).

#### The RFLP of mitochondrial DNA shows lower diversity among the isolates

The digestion of the mitochondrial DNA of the isolates differentiated 11 patterns among the bands larger than 1 kb (Supplementary Fig. S1). The isolates that had identical RAPD patterns did not differ in the mitochondrial patterns either. Overall, the mtDNA RFLP diversity was lower than the RAPD diversity.

#### The PUL4 genes of the isolates also show both intra- and intergenomic diversity

**Figure 3:**
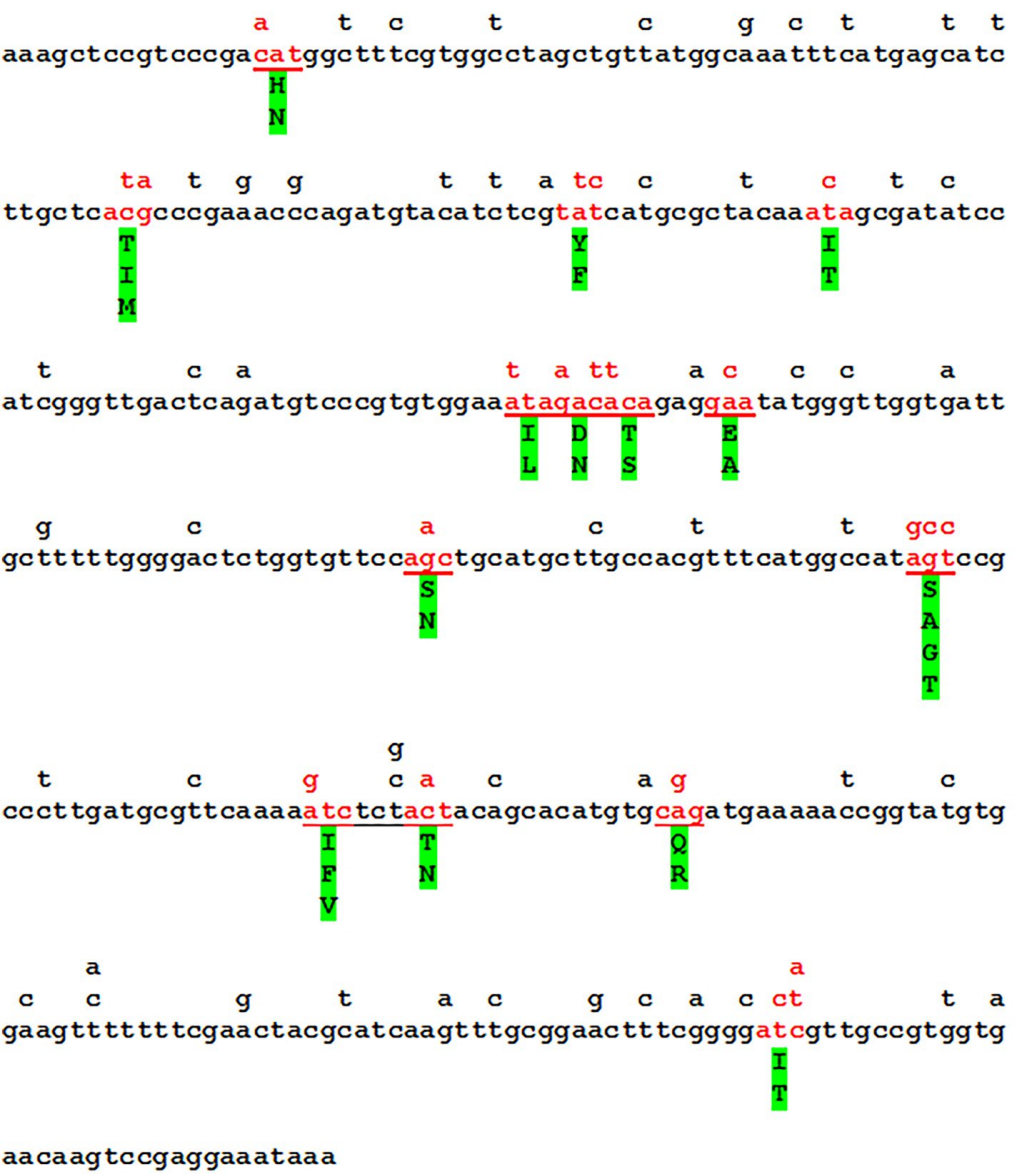
Location of nucleotide substitutions in *PUL4* and their effect on the translated amino acid sequence. The entire sequence of the amplified region extracted from the genome sequences of the type strain of M. pulcherrima is shown. It starts at the 754th nucleotide of the *PUL4* ORF. At each variable position, the nucleotide(s) differing from that of this sequence in certain isolates are shown above it. Where more than one type of SND was found at the site, the number of these nucleotides can be higher than one. All codons in which the nucleotide substitutions caused amino acid substitutions are marked with red character colour. The amino acids that can substitute each other at these sites are shown under the *PUL4* sequence on green background. More specific data are available in Supplementary Table S2.

To find a segment suitable for the examination of the diversity of *PUL4*, the sequences of the *PUL4* genes of the *M. pulcherrima* strains for which genome sequences (GCA_000317355.2; GCA_003017285.1; GCA_003123635.1; GCA_004217705.1; GCA_009746055.1; GCA_009932455.1; GCA_014905795.1; GCA_029606385.1; GCA_030583425.1; GCA_937857175.1) are available in the NCBI Genome database and those cloned from other strains^5^ of the species were aligned. In the MAFFT alignment, a centrally located 380 nt-long segment of the genes proved to be highly variable. This sequence was amplified and sequenced from each isolate. All isolates had ambiguous nucleotides in the sequences, but they differed in the number and location of these nucleotides (Supplementary Table S2; Fig. 3). When all sequences were considered, ambiguous nucleotides were detected in a total of 70 sites (18% of all sites). In all variable positions two nucleotides alternated (SND sites). From the lack of SNP sites it can be inferred that each strain only had two different *PUL4* alleles and thus at least two copies of the *PUL4* gene.

The majority of the dimorphic sites were in third positions of codons in which the nucleotide substitutions did not cause amino-acid differences between the translated sequences (Fig. 3). Translation of all sequences identified altogether 14 codons (12% of all codons) in which the nucleotide substitutions caused amino acid substitutions. Within these codons the first or the second nucleotide position or both positions were variable.

The isolate pairs T15-T16a and B35b-B36b that could not be differentiated by RAPD and mtDNA RFLP analyses were also indistinguishable in *PUL1* heterozygosity but T12a and T12b were differently heterozygous.

### Phenotypic diversity

#### The ability to produce pulcherrimin is variable and prone to reversible segregation

The isolated colonies were maintained on YEA plates. Since the medium was not supplemented with FeCl_3_, all isolates formed faintly pigmented colonies. To compare their capacity to produce pulcherrimin, the isolates were re-inoculated onto plates supplemented with various concentrations of FeCl_3._ Both the intensity of colony colour and the formation of pigmented halo in the agar medium around the colonies were compared. The isolates were diverse in both traits, but the colony colour increased and the size of pigmented halo decreased with the increase of the iron content in the medium (Supplementary Table S3). The halos were only faintly visible on YEA plates without FeCl_3_ supplementation, so their width could not be measured.

**Figure 4:**
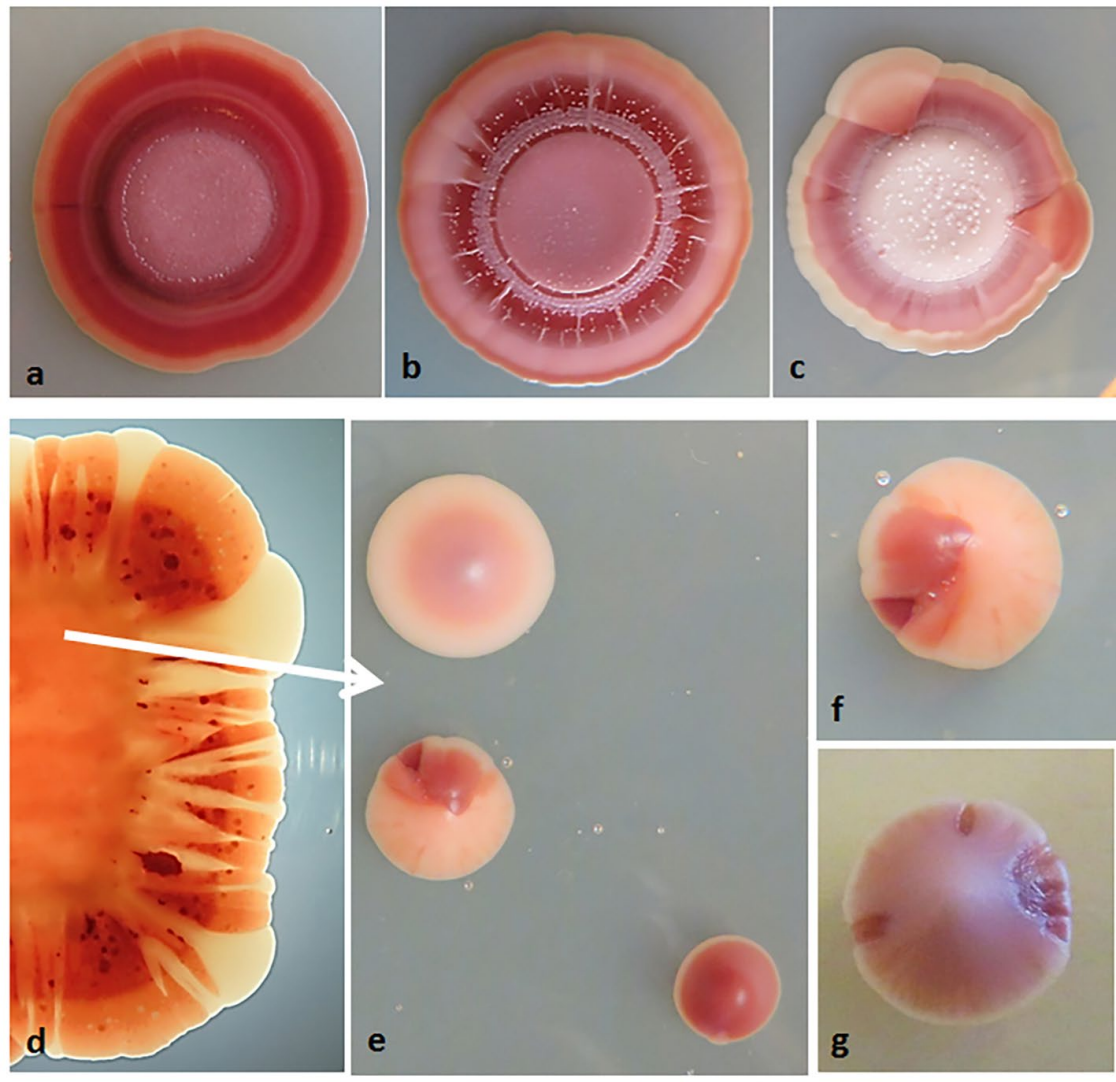
Pulcherrimin production. Colony morphology on YEA supplemented with 0.04 mg/ml FeCl_3_. Colonies formed from dropped suspesions of the isolates B19b (**a**), T16a (**b**) and T12a (**c**). A colony formed from cells of B1 smeared on the medium (**d**). Colonies formed by cells of D spread on the medium (**e** and **f**). A segregating colony of B19 (**g**).

Upon longer periods of incubation (3 weeks or more), sectors of various colour intensities appeared in the colony edges of certain isolates (Fig. 4, Supplementary Table S3). This observation hints at instability (segregation) in pulcherrimin production. When diluted samples of suspensions of these colonies were spread onto plates with iron supplementation, mixed populations of single-cell colonies of different colour intensities were formed. However, not all colonies were homogeneously coloured or white. There were also colonies containing darker or fainter sectors, indicating that the segregation was reversible.

#### The strength of antagonism is also diverse

**Figure 5:**
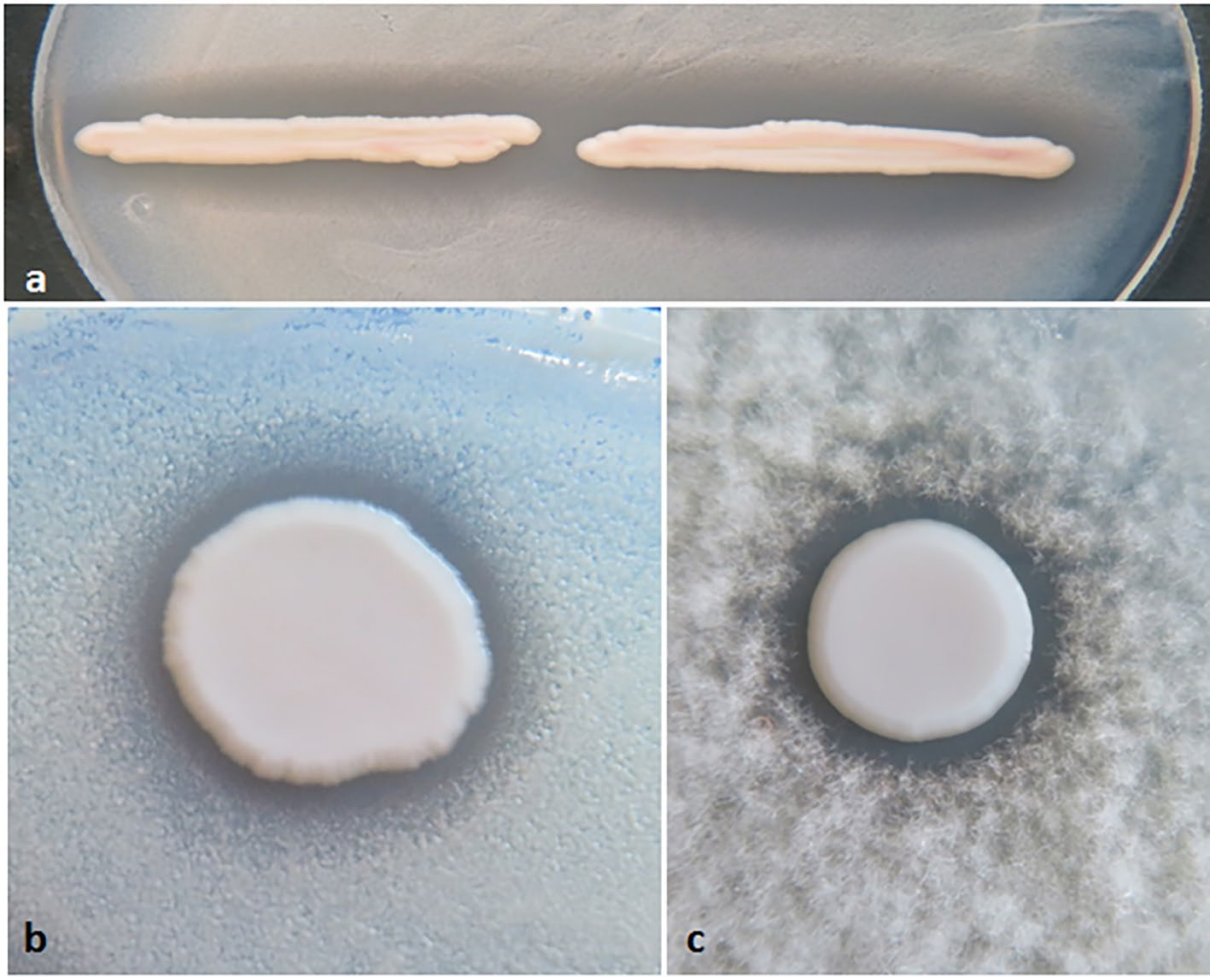
Antifungal antagonism. Inhibition zones around four isolates in the YOI (Yeast on indicator) test. (**a**) Isolates Q14 (left) and T67 (right) on *Zygosaccharomyces* lawn. (**b**) Isolate B1 on *Debaryomyces* lawn. (**c**) Isolate B19b on a lawn of *Botrytis* conidia.

As the ability to produce pulcherrimin correlates with the ability to antagonise other microorganisms^12^, the isolates were also tested for antifungal activity with three “indicator” organisms on two types of media that were not supplemented with FeCl_3_. All isolates proved to be antagonistic against *Zygosaccharomyces* and *Botrytis* and most of them could also inhibit the growth of *Debaryomyces* but less efficiently (Supplementary Table S3; Fig. 5). Remarkably, no clear correlation was seen between the presence of pigmented halos on media supplemented with iron and the antagonistic efficiency on media with no iron supplementation.

#### All isolates can switch to invasive pseudohyphal growth but vary in the efficiency, morphology and stability of this trait

**Figure 6:**
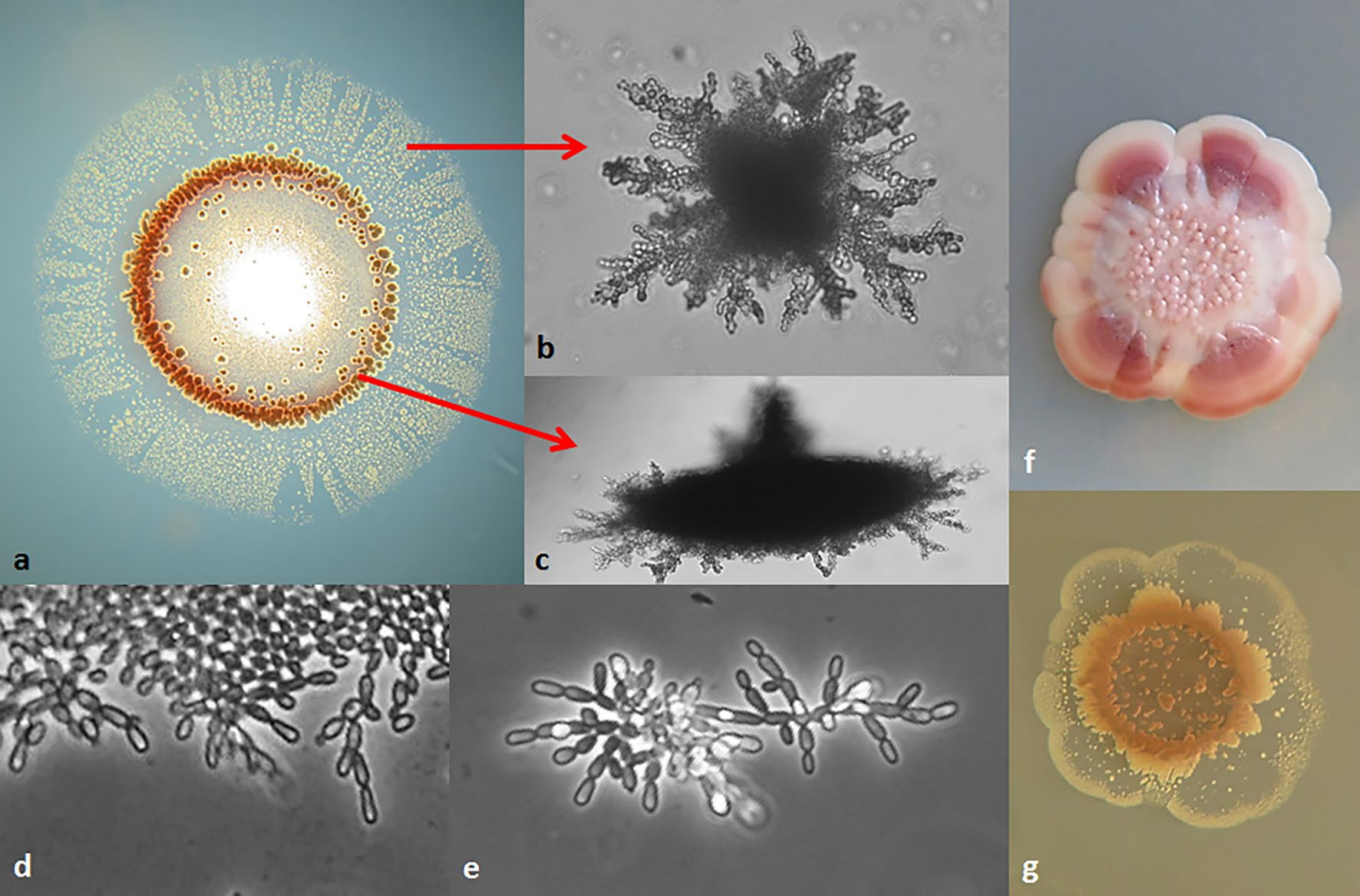
Substrate invasion. (**a**) Transilluminated image of the invasive growth (intrusions) of a colony of the isolate A59 after washing off the cells from the surface of the agar medium. (**b** and **c**) Two morphological types of the intrusions. (**d**) Formation of pseudohyphae at the edge of a culture growing in a sandwich culture. (**e**) Structure of a pseudohypha formed in a sandwich culture. (**f**) Sectors of various pigmentation intensity on the surface and (**g**) invasive growth into the medium in a colony of the isolate B58.

As substrate colonisation through the formation of invasive pseudohyphae was observed in certain *M. pulcherrima* strains in previous studies^8^, the isolates were also tested for the ability to grow into the agar medium. All isolates were able to penetrate the medium but with highly diverse efficiency and morphology (Supplementary Table 3S; Fig. 6; Supplementary Fig. S2). Invasive growth was mainly visible under the edges of the colonies and under the circumferential “extension belt” formed by cell propagation at the colony edges (Fig. 6a). Under the edges usually dense spindle-like bodies were formed from which pseudohyphae emanated (Fig. 6c). Under the extension belts, detached patches of looser pseudomycelium were formed (Fig. 6d). The isolates varied in the size, density and morphology of both types of intrusion. Morphological variation was frequently seen even within the colonies. Different patches formed sectors (Fig. 6a, Supplementary Fig. S2) similar to the differently coloured sectors observed in the pulcherrimin tests. However, the differently coloured sectors did not correlate with the differently invasive sectors (Fig. 6f and g). The structure of the invasive pseudomycelium could not be examined microscopically because of the blurring effect of the medium. For microscopic examination the sandwich method was used. The formation and extension of the pseudohyphae was monitored in thin agar layers sandwiched between microscopic slides and cover slips, where the oxygen supply was limited as in the medium during invasive growth. These pseudohyphae exhibited the standard morphology of rudimentary pseudohyphae: elongated unipolarly budding cells connected by uncleaved septa between the mother and daughter cells (Fig. 6d and e).

#### Chlamydospore formation is less diverse

A characteristic feature of *M. pulcherrima* is the production of chlamydospores, large globouse cells containing large lipid globules^8,22,23^). A recent study observed a positive correlation between the relative number of chlamydospores in the culture and the intensity of pulcherrimin production^24^. Prompted by this observation, the efficiency of chlamydospore formation and the colour intensity of the colonies of the isolates were compared. The percentage of chlamydospores varied in the range of 0–25% (Supplementary Table S3, Supplementary Fig. S3), but correlated neither with pigmentation intensity nor with antagonistic efficacy.

## Discussion

Amplicon sequencing identified very high numbers of (predominantly dimorphic) ambiguous nucleotide positions in the primary barcode segments (D1/D2 and ITS1-5.8S-ITS2) of all new isolates. Previous studies revealed similar nucleotide ambiguity in the type strains of the pulcherrimin-producing species recently merged in *M. pulcherrima*^5,6^. In addition to their internal diversity the new isolates and the type strains of the former species also differed from each other in the number and location of the ambiguities. These results demonstrate that very high intragenomic and intergenomic variability is characteristic of the repeats coding for rRNA in *M. pulcherrima*, much higher than in any other yeast species for which data are available.

The distribution of the ambiguous sites differed in the two barcodes. Whereas in D1/D2 the SND sites occurred in four short segments, they were randomly scattered across ITS1. This difference can be attributed to different roles of the transcripts in the generation of rRNA for the ribosomes. The former is part of the large subunit rRNA whose correct folding is essential for the ribosome. Consistent with previous observations made in the type strains of the former species^5^, SNDs were found in the D1 and D2 hairpin loops (stem loops) of the reconstructed secondary RNA structures. From these specific locations, it can be inferred that nucleotide substitutions are not allowed in segments important for correct RNA folding but can be tolerated in structurally less critical (double-helical) segments. The double helical segments are less sensitive to substitutions because of the neutralising effect of wobble pairing with the nucleotides with the other strand of the loop^5^. Because of the concentrated occurrence of the SND sites, large parts of the D1/D2 barcodes are free of ambiguity and thus suitable for the determination of the isolates’ taxonomic affiliation. The transcripts of the ITS segments are not so structure-sensitive because they are not incorporated in the ribosomes. The extremely high numbers of di- and polymorphic sites and the deletions of various lengths in ITS2 make this barcode unsuitable for taxonomic identification of isolates in this group of organisms. This is another peculiarity of *M. pulcherrima* because it is the ITS region that was proposed as the universal barcode marker for Fungi^25^. It should be noted that the analysis of ITS sequences did not give clear results in numerous other yeast groups either, therefore the D1/D domain has become preferentially used in yeasts^26^. The results of this study are in line with this practice.

Remarkably, the intragenomic barcode variance (the proportion of di- and polymorphic sites) was much greater in all isolates than the gaps separating many ascomycetous species and even certain related genera^27^, as if these isolates had accumulated barcodes from several species. This phenomenon may cause false overestimation of taxon diversity in metabarcoding and metagenomic analysis of environmental samples, in which the delimitation of OTUs (species-like units) is based on (arbitrarily) set similarity thresholds (e.g. 97%). When a strain has barcode segments that are less similar than the threshold, the analysis may consider them representatives of different OTUs (species). The high number of ambiguous nucleotides in barcode sequences obtained by direct amplicon sequencing may cause difficulties in patenting of biotechnological strains. *M. pulcherrima* strains are often employed as biological control agents [BCAs] in the management of crop pathogens or as lipogenic/oleaginous yeasts^8^.

Obviously, the *M. pulcherrima* repeats coding for rRNAs evolve in a non-standard way. In a previous study the intragenomic diversity of the primary barcodes of a *M. fructicola* (synonym of *M. pulcherrima*) strain was proposed to be due to the evolution of the repeats by a birth-and-death-type mechanism instead of homogenisation operating in other yeasts^7^. In yeasts, a similar mode of repeat evolution was only observed in *Cyberlindnera* but with much lower intragenomic repeat diversity^28^. In an earlier study^29^, different D1/D2 sequences were detected in certain *Clavispora lusitaniae* strains. Their presence within the same genome may be due to birth-and-death evolution, but this possibility was not considered.

The analysis of 33 protein-encoding nuclear genes (including secondary barcode segments) performed in recent studies^5,6^ revealed chimeric genome structures in the type strains of the merged species. The analysed genes usually had two or more copies that differed in sequence and phylogenetic history. The four genes of the *PUL* cluster were among them. Most strains involved in that analysis had two sets of *PUL* genes. The sets often differed from each other more than from one or the other set of a different strain (higher intragenomic than intergenomic differences). Here *PUL4*, as the representative of the *PUL* cluster was used to get an insight into the genome structures of the isolates. The sequences of the amplified *PUL4* segments had multiple dimorphic sites in all isolates, demonstrating that these isolates also had at least two different *PUL4* genes. Thus, the intragenomic sequence diversity is not confined to the primary barcodes. Most SNDs occurred in the third positions of codons and had no effect on the amino acid sequences of the proteins. The nucleotide substitutions in the first and second codon positions caused amino acid substitutions in altogether 12% of the codons. It can be inferred from these results that the different *PUL4* genes within a genome are orthologues of different origin that were brought together by hybridisation. The *PUL4* sequences (including the ambiguous sites) also showed considerable interstrain (intergenomic) variations. The interstrain differences indicate that the strains of the species may have chimeric genome structures. The RAPD patterns covering entire genomes and the RFLP patterns of the mitochondrial genomes also revealed high interstrain differences which also proves that *M. pulcherrima* is a very heterogeneous species.

To examine whether the genomic/genetic differences are reflected in the phenotypes of the isolates, three properties relevant for the application of *M. pulcherrima* strains as bioprotective agents were examined: iron-chelating efficiency (intensity of pulcherrimin production), antifungal antagonism (inhibition of tester indicator organisms) and substrate invasion (transition to invasive pseudohyphal growth). The former two properties are assumed to be linked because the synthesis of pulcherriminic acid is involved in both. The higher the pulcherrimin production, the stronger the antimicrobial activity^12^. Considerable diversity was found in both of them, but the results of the antagonism tests carried out on different culture media and with different indicator organisms were not completely congruent. The lower inhibition efficiency against *Debaryomyces* can be attributed to the higher growth vigour (faster growth) of this yeast. The more efficient inhibition of *Botrytis* can be due to the higher vulnerability of the germination of conidia compared to that of the budding of yeast cells.

The efficiency of the yeast-to-pseudohypha transition and the morphology of substrate invasion by the pseudomycelium also greatly varied among the isolates. Switching from the planktonic yeast growth to the formation of chains of non-separated cells (pseudohyphae) may play an important role in the protection of the plant surface because the pseudomycelium formed by the invasive pseudohyphae can form biofilms on the lesions which are also gateways for the invasion by destructive microorganisms. Previous antagonism studies tested the ability of *Metschnikowia* (planktonic) yeast cells for adhesion to non-plant surfaces such as glass and polypropylene^21^. The ability of the tested strains to form pseudohyphae was not examined, although a pseudomycelium is a stronger structure than a layer of planktonic yeast cells, especially if its pseudohyphae establish a strong bond with the damaged plant tissue by penetrating it.

In addition to these three traits, chlamydospore formation, a trait not known to have relevance to antagonism, was also examined. The isolates also varied in the efficiency of chlamydospore formation but the overall diversity of this trait was lower.

When the molecular differences were compared with the phenotypic differences, no clear correlation between the examined genetic/genomic diversities and the phenotypic diversities was noticed. Thus, none of the molecular tests employed in this study can be used for differentiation of strains showing different phenotypes.

Interestingly, pulcherrimin production and pseudohyphal growth also varied within clones (single-cell colonies) of numerous isolates. Intraclonal changes (segregation) of the intensity of pulcherrimin production in *M. pulcherrima* were already reported in previous studies and found reversible^6^. The cultures of numerous new isolates described in this study also formed sectors differing in colour intensity and mixtures of differently coloured single-cell colonies. The colonies often had sectors differing in colour from the rest of the colony. Thus, the changes (segregation) of the intensity of pulcherrimin production were reversible in these isolates as well. These changes may be due to epigenetic processes (silencing and reactivation of regulators) rather than to mutations and backmutations. The intraclonal heterogeneity of pseudohyphal growth may be attributed to similar processes. However, it has to be mentioned that in *Saccharomyces cerevisiae,* whose epigenetic processes have been most deeply explored among yeasts, certain important processes such as DNA methylation, repressive histone methylation, and siRNAs may be missing^30^. Experiments are already underway to learn more about the reversible transitions in *M. pulcherrima* strains and explore the underlying mechanisms.

## Methods

### Sample collection and yeast isolation

Overripe and fallen fruits were collected from abandoned roadside and orchard trees or purchased from greengrocers. Decaying tissue blocks were cut from them for yeast isolation. The blocks were homogenised in sterile water and loopful amounts of the homogenised material were spread onto YEA (Yeast Extract Agar: 0.5% yeast extract, 2% glucose, 2% agar) plates supplemented with 0.04 mg/ml FeCl_3_. Colonies producing pulcherrimin turn red on this medium. After 7 days of incubation at 25 °C, 1 to 2 red-brownish colonies were isolated from each sample (Supplementary Table S1) and inoculated onto YEA plates for maintenance.

### Barcode amplification and sequencing

DNA was isolated from overnight cultures grown in YEL broth (YEA without agar) and the isolated DNA was used for amplification of chromosomal segments. The D1/D2 domains and the ITS1-5.8S-ITS2 segments of the repeats encoding rRNA were amplified with the primer pairs NL1-NL4^31^ and ITS1-ITS4^32^), respectively. The amplified fragments were sequenced in both directions using the same primer pairs. The D1-D2 sequences were deposited in GenBank (https://www.ncbi.nlm.nih.gov/genbank/) under accession numbers listed in Supplementary Table S1. The ITS sequences could not be deposited in the database because of the very high number of ambiguous nucleotides.

### Amplification and sequencing of *PUL4*

*PUL4* is one of the four genes of the *PUL* cluster. It codes for a putative transcription factor belonging to a subclass of zinc finger proteins found exclusively in fungi. The Pul4 proteins contain a Fungal_TF_MHR region, a fungal transcription factor regulatory middle homology region^33^. A 380 nt-long segment of this region was amplified from the DNA prepared for barcode sequencing with the primer pair PUL4F (742–767: CCCAGCATGTTGAAAGCTCCGTCCCG) and PUL4R (1115–1144: AGTAATTGGTAGTTTATTTCCTCGGACTTG). The amplicons were sequenced with the same primers and the sequences were deposited in GenBank. The accession numbers are listed in Supplementary Table S1.

### Sequence analysis

To identify variable positions, the nucleotide sequences were aligned with the MAFFT version 7 multiple alignment algorithm available at https://www.ebi.ac.uk/jdispatcher/msa/mafft^34^. The *PUL4* sequences were also translated using the NCBI ORF finder (https://www.ncbi.nlm.nih.gov/orffinder/) and the amino acid sequences of the Pul4 proteins of the isolates and those extracted from the whole-genome sequences of *M. pulcherrima* strains available in the NCBI genome database (https://www.ncbi.nlm.nih.gov/genome/?term=) were aligned with the same MAFFT tool. Minimum free energy secondary structures were generated for the D1 and D2 loops of the transcripts of the D1/D2 domains with Predict, a secondary structure webserver **(**http://rna.urmc.rochester.edu/RNAstructureWeb/Servers/Predict1/Predict1.html**)** using default settings.

### RAPD-PCR

Samples of the solutions of genomic DNA (50 ng DNA) were used for RAPD-PCR reactions with the primers RAPD24 (5ʹ-GCGTGACTTG-3ʹ)^35^ and RAPD1283 (5ʹ-GCG ATC CCC A-3ʹ)^36^ and GoTaq DNA polymerase. The PCR reactions were performed with the following programme: 94 °C for 5 min, 30x (94 °C for 50 s, 38 °C for 50 s, 72 °C for 50c), 72 °C for 5 min. The amplified fragments were separated in 1.2% (w/v) agarose gels (1× TBE buffer) stained with ethidium-bromide. The band patterns were analysed using the GelAnalyzer 2010 software^37^ with manual adjusting and the results were converted to binary sets of data. The data sets were subjected to cluster analysis by the UPGMA (unweighted pair group method with arithmetic means) algorithm using the version available at http://genomes.urv.es/UPGMA^38^.

### Mitochondrial DNA extraction and restriction analysis

Mitochondrial DNA was prepared from exponential-phase YEL cultures with the method described in ^39^ and digested with *Mbo*I. The fragments were separated by electrophoresis in 0.7% agarose, 0.5× TBE. The band patterns were analysed as described for RAPD-PCR.

### Examination of pulcherrimin production

Pulcherrimin production was examined on YEA plates supplemented with 0.02 mg/ml and 0.04 mg/ml FeCl_3_. Colonies producing pulcherrimin turn red on these media. The higher the concentration of FeCl_3_, the more intense the pigmentation. When pulcherrimin is also produced extracellularly, a coloured halo is formed around the pigmented colony. The isolates were inoculated on these plates in two ways, either by streaking (smearing) cells with a loop or by dropping 10 μl of dense suspension of cells (10^7^ cells/ml). Growth and pigment formation were monitored at 25 °C for four weeks. With this method the stability of pulcherrimin production could also be tested because the colonies of the unstable isolates formed sectors of different colour intensities at their edges. Stability was also tested by spreading cells on an iron-containing medium and monitoring the formation of sectors within colonies growing from single cells.

### Examination of chlamydospore formation

This trait was examined in cultures grown on vegetable juice medium prepared from diluted (1:20) BIO Gemüsesaft (Josef Pölz, Bio-Produkte, 84,518 Garching an der Alz, Germany) and supplemented with 2% agar^7^. 10-μl aliquots of dense suspensions of cells (10^7^ cells/ml) were dropped onto the medium and incubated at 14 °C. After 2 weeks of incubation, samples of the cultures were examined microscopically.

### Invasive growth and formation of pseudohyphae

To test the isolates for the ability to penetrate the agar medium, 10-μl samples of dense cell suspensions (10^7^ cells/ml) were dropped onto YEA. After two weeks of incubation at 25 °C, the colonies were washed off from the plate with water to visualise the intrusions into the agar medium beneath them. The morphological structure of the intrusions was examined microscopically at low magnification. As the microscopic images were blur because of the distorting effect of the agar medium, the transition from the yeast growth to the invasive filamentous growth was also examined in thin films (0.5–1 mm) of YEA sandwiched between microscope slides and coverslips as described previously^40^.

### Antagonism tests

The isolates were tested for antimicrobial antagonistic activity by the “Yeast on indicator (YOI)” method^41^. Briefly, the agar test plate was flooded with a dense suspension (10^7^ cells/ml) of cells or conidia of the indicator organism to obtain a homogeneous lawn on the surface of the medium. After removing the rest of the suspension and drying the surface of the plate, loopful amounts of the *Metschnikowia* isolates were smeared on the lawn to form spots of ~ 5 mm in diameter. The growth of the spots and the lawn around them was monitored at 20 °C for 7 days. If the isolate inhibited or reduced the growth of the lawn, a clear (or only turbid) inhibition zone appeared in the lawn around its colony. The tests were carried out on YEA and EMMA (Edinburgh Minimal Medium,^42^) plates and the indicator organisms were *Debaryomyces* aff. *hanseni* Vm7^43^, *Zygosaccharomyces* aff. *siamensis* 11-2106 and *Botrytis cinerea* 3318^12^.

### Supplementary Information

Supplementary Information.

## Data Availability

The datasets analysed during the current study are included in this published article [and its supplementary information files]. The D1/D2 and *PUL4* sequences are available in GenBank. The GenBank accession numbers of are listed in Supplementary Table S1.
