# Supplementary material for: High intragenomic, intergenomic, and phenotypic diversity in pulcherrimin-producing Metschnikowia yeasts indicates a special mode of genome evolution
Source: Sci Rep. 2024 May 8;14:10521. doi: 10.1038/s41598-024-61335-5 (PMC11076541; doi:10.1038/s41598-024-61335-5)
Supplement: Supplementary file 1 — Supplementary Information. [file 41598_2024_61335_MOESM1_ESM.pdf]

High intragenomic, intergenomic, and phenotypic diversity in pulcherrimin producing *Metschnikowia* yeasts indicates special mode of genome evolution

Matthias Sipiczki[1*], Kinga Czentye[1], Zoltán Kállai[1,2]

[1]Department of Genetics and Applied Microbiology, University of Debrecen, Debrecen, Hungary
[2]Present address: Institute of Horticulture, University of Debrecen, Debrecen, Hungary
*corresponding author: gecela@post.sk

This file includes:

Supplementary Tables S1-S3
Supplementary Figures S1-S3
Full-length gel image for Fig. 2.

Supplementary Table S1. List of isolates and GenBank accession numbers of sequences

Isolate	Source of sample		Accession number	
	Substrate	Collected in	D1/D2	*PUL4*
A2	apple	Békéscsaba, Marasť	OR826398	PP100800
A59	apple	Zelemér	OR826399	PP100801
A60	apple	Zelemér	OR826401	PP100802
B1	pear	Gerendás, Ulička	OR826402	PP100804
B19a	pear	Gerendás, Ulička	OR826403	PP100805
B19b	pear	Gerendás, Ulička	OR826606	PP100806
B20a	pear	Zelemér	OR826605	PP100807
B20b	pear	Zelemér	OR826609	PP100810
B21	pear	Zelemér	OR826611	PP100811
B22	pear	Zelemér	OR826610	PP100812
B35a	pear	Debrecen, Biczó kert	OR826629	PP100813
B35b	pear	Debrecen, Biczó kert	OR826631	PP100813
B36a	pear	Debrecen, Biczó kert	OR826634	PP100815
B36b	pear	Debrecen, Biczó kert	OR826635	PP100816
B52	pear	Bodaszőlő	OR826653	PP100817
B53	pear	Bodaszőlő	OR835226	PP100818
B54	pear	Bodaszőlő	OR835228	PP100819
B57	pear	Bodaszőlő	OR835232	
B58	pear	Bodaszőlő	OR835235	PP100820
T4a	grape	Békés, cimitir	OR835449	PP100821
T4b	grape	Békés, cimitir	OR835451	PP100822
T5	grape	Békés, cimitir	OR835452	PP100823
T12a	grape	Gerendás, Ulička	OR835453	PP100824
T12b	grape	Gerendás, Ulička	OR835459	PP100825
T15	grape	Békéscsaba, Marasť	OR835806	PP100826
T16a	grape	Békéscsaba, Marasť	OR835807	PP100827
T16b	grape	Békéscsaba, Marasť	OR835814	PP100828
T40	grape	Debrecen, Kerekestelep	OR835815	PP100829
T62	grape	Bodaszőlő	OR835816	PP100830
T63	grape	Bodaszőlő	OR835818	PP100831
T64	grape	Bodaszőlő	OR835824	PP100832
T65	grape	Bodaszőlő	OR835835	PP100833
T66	grape	Bodaszőlő	OR838683	PP100834
T67	grape	Bodaszőlő	OR835834	PP100835
Q14	quince	Debrecen, Kerekestelep	OR835236	PP100803
Z17	Khaki plum	Békéscsaba, grocery	OR835838	PP100808
Z18	Khaki plum	Békéscsaba, grocery	OR835841	PP100809

Supplementary Table S2. Variable nucleotide positions in the sequenced *PUL1* segments and their effect on the amino acid sequences. Nucleotides of dimorphic positions with effect on the amino acids are marked with blue background. [1] In the genome sequence of the *M. pulcherrima* type strain CBS 5833^T (NRRL Y-7111^T). [2] Available in databases. SND symbols: K: G or T (transversion), M: A or C (transversion), R: A or G (transition), S: C or G (transversion), Y: C or T (transition), W:A or T (transversion).

Position in the gene sequence[1]	Nucleotides in genes of genome sequences and in cloned sequences[2, 3]	In isolates		
		Nucleotides[3]	Possible intragenomic combinations of codons coding for different amino acids	Amino acid substitutions
769	A, C	C or M(A/C)	CAT or AAT OR CAT/AAT	H or N or H/N
774	A, C, T	T or Y(C/T)		
777	C, T	C or Y(C/T)		
783	C, T	C or Y(C/T)		
792	C. T	T or Y(C/T)		
798	A	A or R(A/G)		
801	T	T or Y(C/T)		
804	C, T	C or T or Y(C/T)		
810	C, T	C or T or Y(C/T)		
813	C, A	C or Y(C/T)		
821	C	C or Y(C/T)	ACG or ATG or ACA or ATA or ACG/ATG or ACG/ACA or ACG/ATA etc.	T or I or M or T/I or T/M or I/M
822	G	G or R(A/G)		
825	C	C or Y(C/T)		
828	A	A or R(A/G)		
831	C, G	C or S(C/G)		
840	C, T	C		
843	C, T	C		
846	G	G or R(A/G)		
848	A	A or W(A/T)	TAC or TTC or TAC/TTC	Y or F or Y/F
849	C, T	C		
852	T	T or Y(C/T)		
858	A, C	A or W(A/T)		
863	T	T or Y(C/T)	ATA or ACA or ATA/ACA	I or T or I/T
867	G	G or K(G/T)		
870	A, C	A		
878	C	C or Y(C/T)		
885	A, C, T	C or T or Y(C/T)		
888	A, G	A or G or R(A/G)		
904	A, T	A or W(A/T)	ATA or TTA or ATA/TTA	I or L or I/L
906	A, G	A		
907	A, G	G or A	GAC or GAT or AAC or AAT or GAC/GAT or GAC/AAC etc.	D or N or D/N
909	C	C or Y(C/T)		
910	A	A or T or W(A/T)	ACA or TCA or	T or S or

			ACA/TCA	T/S
915	A, G	G or R(A/G)		
917	A, G	A or M(A/C)	GAA or GCA or GAA/GCA	A or E or A/E
921	T	T or Y(C/T)		
924	G	G or S(C/G)		
930	A, G	G		
936	G, T	T or K(G/T)		
945	A, C	A or C or M(A/C)		
959	G	G or R(A/G)	AGC or AAC or AGC/AAC	S or N or S/N
969	C, T	C or Y(C/T)		
975	G, T	G or T or K(G/T)		
984	C, T	C or Y(C/T)		
988	A, G	A or G or R(A/G)	AGT or AGC or ACC or ACT or GCC or GGC or GCT or AGT/AGC or AGT/ACC, etc.	S or A or G or T or S/A or S/G, etc.
989	C, G	C or S(C/G)		
990	C, T	T or C		
996	C	C or Y(C/T)		
1005	C, T	C or T or Y(C/T)		
1012	A, T	A or G or R(A/G)	ATC or GTC or ATC/GTC	I or V or I/V
1015	A, T	T		
1017	G, T	G or T or K(G/T) or S(C/G) or Y(C/T)		
1019	C	C or M(A/C)	ACT or AAT or ACT/AAT	T or N or T/N
1023	A	A, M		
1032	A, G	A or G or R(A/G)		
1034	A	A or R(A/G)	CAG or CGG or CAG/CGG	Q or R or Q/R
1044	C, T	C or Y(C/T)		
1050	C, T	T		
1055	A	A or M(A/C)		
1059	A, C, T	A or C or T or W(A/T)		
1068	A, G	A or R(A/G)		
1074	C	C or T or Y(C/T)		
1080	G	G or R(A/G)		
1083	C, T	C or T or Y(C/T)		
1098	A, G	A or R(A/G)		
1092	C, T	C or T or Y(C/T)		
1095	A, G	G		
1098	A, G	G or S(C/G)		
1100	T	T or Y(C/T)	ATC or ACC or ACT or ATT, ATA or ATC/ACC or ATC/ACT, etc.	I or T or I/T
1101	C, T	C or T or M(A/C) or Y(C/T)		
1110	G, T	G or T or K(G/T)		
1113	A, G	A or G or R(A/G)		

Supplementary Table S3. Phenotypic properties of isolates. (.): thin sharp line of inhibition around the edge of the indicator colony. Vm7: *Debaryomyces* aff. *hanseni*. 11-2106: *Zygosaccharomyces* aff. *siamensis.*

Isolate	Pulcherrimin production on YEA					Chlamydospores (%) on Vegetable agar	Inhibition zone (width in mm) on				Invasive growth on YEA	
	Colony colour		Pigmented halo		Segregation		YEA	EMMA			Intensity	Morphology
	0.02 mg/ml $FeCl_3$	0.04 mg/ml $FeCl_3$	0.02 mg/ml $FeCl_3$	0.04 mg/ml $FeCl_3$	0.04 mg/ml $FeCl_3$		Yeasts			*Botrytis*		
							Vm7	Vm7	11-2106			
A2	++ s	++	(+)	-	+	-	0.5	1	1.5	2	++	
A59	++ s	++	-	-	+	5	(.)	0.5	1	2	+++	sectored
A60	++	++	-	-	+	5	0.5	2	1.5	2	+++	
B1	++	++	++	++	+	5	2	2	3	2	(+)	
B19a	+(+)	++	+++	++	+	1	1.5	1	3	2	++	sectored
B19b	+(+)	++	-	-	-	1	1.5	1.5	5	3	++	sectored
B20a	++	++(+)	++	+(+)	-	-	0.5	-	2	2	+(+)	
B20b	++	++(+)	++	+(+)	-	5	-	0.5	5	1.5	+	
B21	(+) s	+ s	-	-	-	5	1	-	3	1	(+)	
B22	(+)	+(+)	+	-	-	-	1.5	1	5	3	-	
B35a	++ s	++	-	-	+	-	(.)	-	4	2	++	sectored
B35b	++ s	++	-	-	+	<1	-	-	3	2	++	sectored
B36a	++	++	+(+)	-	-	5	1	(.)	3	2	+	
B36b	++	++	-	-	+	1	-	-	2	2	++(+)	
B52	+	++	-	-	-	10	.	-	2	1.5	+(+)	
B53	+ s	++	-	-	-	1	1	1	2	1.5	+++	
B54	++	++	-	-	+	20	1	1	2	1	++	
B57	+	++	-	-	-	21	(.)	(.)	2	2	+++	undulate
B58	++	++	-	-	-	-	0.5	0.5	1	1.5	++	undulate
T4a	++	++	++	+	-	-	2	3	2	1	++	undulate
T4b	+	++	-	-	-	1	2	2	4	2	+++	sectored
T5	+	+	+	-	-	<1	2	2	1	1.5	+	

T12a	+	+	-	-	+	5	(.)	1	4	2	++	undulate
T12b	+	+++	-	-	+	1	-	1	3	2	++	
T15	++	+++	+(+)	+	-	-	2	3	3	2	++	sectored
T16a	++	+++	+	(+)	-	<1	2	3	3	2	+(+)	
T16b	++	+++	+	(+)	+	1	(.)	1.5	3	2.5	+++(+)	
T40	+(+)	++(+)	+	(+)	-	5	(.)	1	3	2.5	+	
T62	++	++	(+)	-	-	1	0.5	1.5	2	2.5	+	
T63	++(+)	+++	(+)	-	-	-	1	1	4	2	+	
T64	++(+)	+++	-	-	-	2	(.)	0.5	1	1.5	(+)	
T65	+++	++++	-	-	-	-	1	1	2	3	+(+)	
T66	++	++	-	-	-	1	1	1	2	2	++	
T67	++	++	-	-	-	3	2	1	10	1.5	++	
Q14	+	+(+)	-	-	-	5	(.)	0.5	8	1.5	++	undulate
Z17	++	+(+)	-	-	+	1	(.)	(.)	2	1	+++	
Z18	++ s	+(+)	-	-	+	1	1.5	2	2	1	++(+)	

a

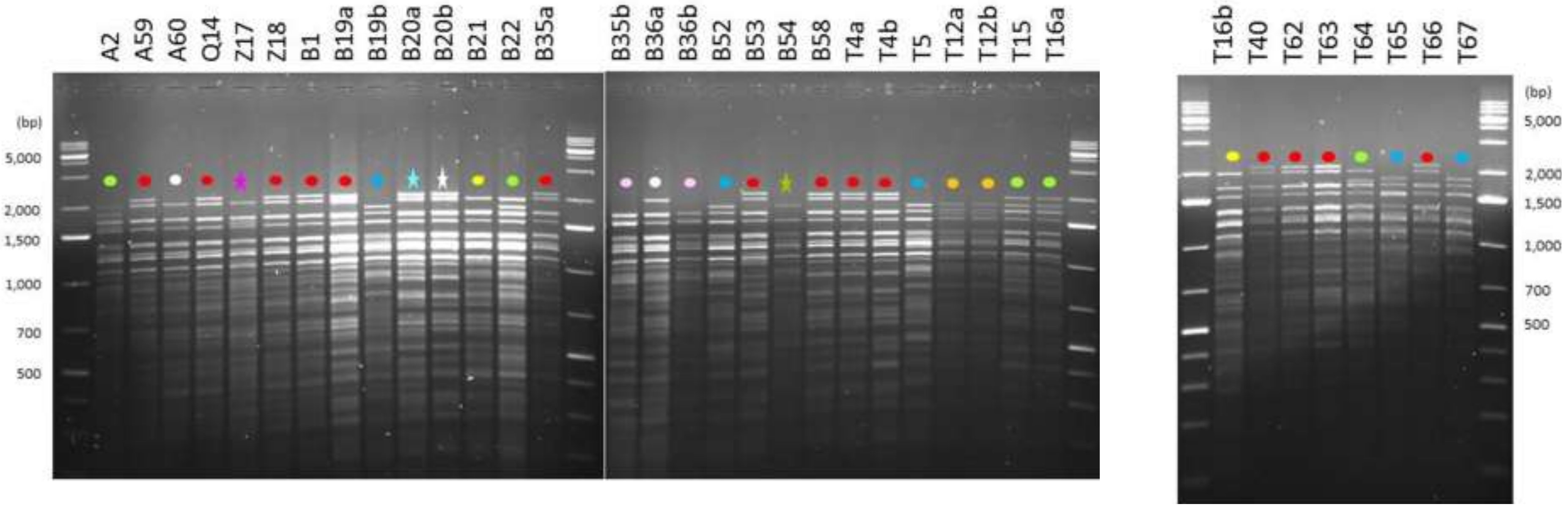

b

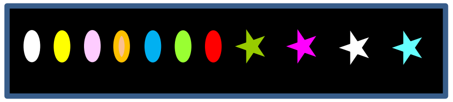

Supplementary Figure S1. RFLP analysis of the mitochondrial genomes. **a**. Banding patterns of the new isolates. Different patterns are marked with different symbols. **b**. List of patterns.

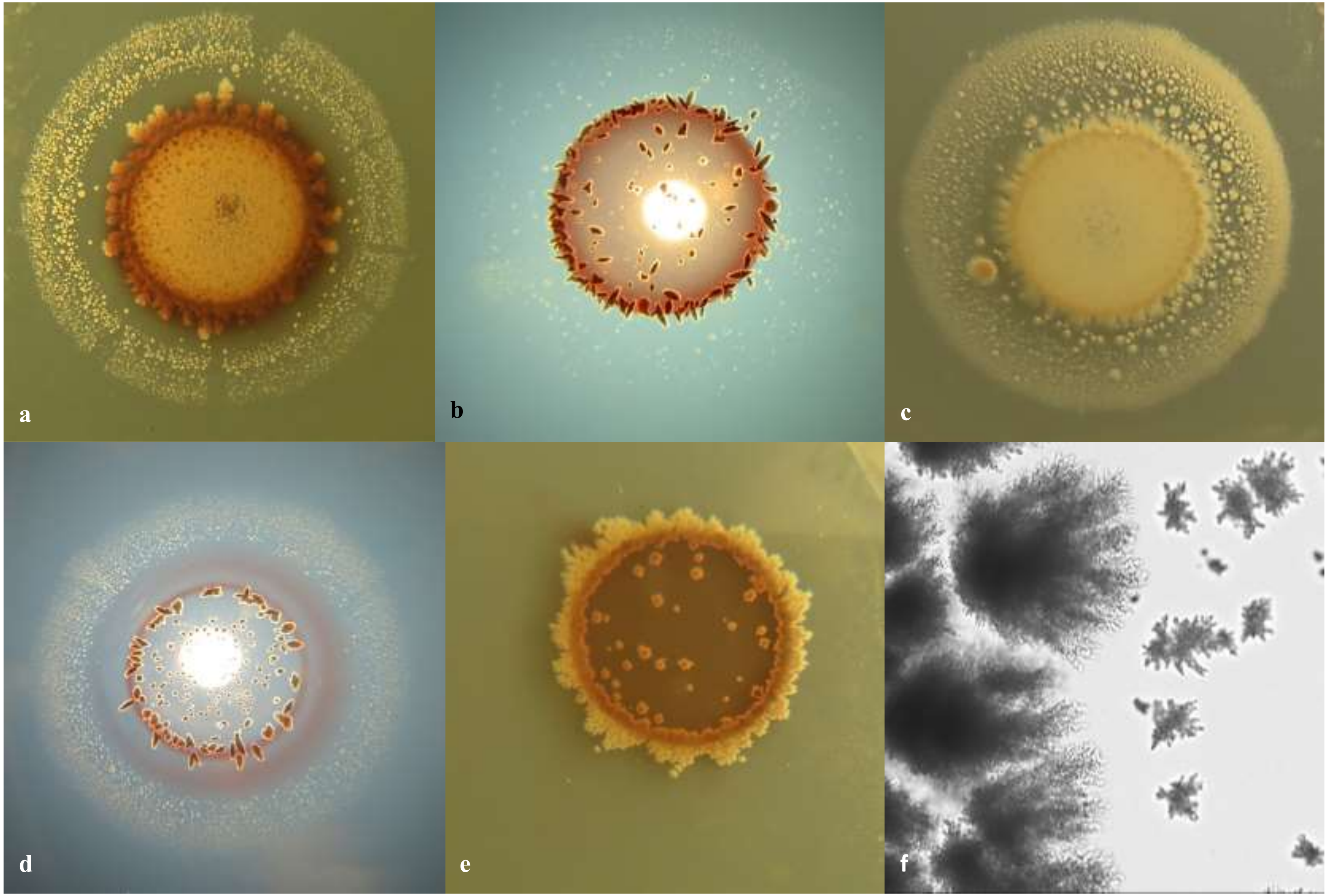

Supplementary Figure S2. Substrate invasion. Intrusion into the agar medium under the colonies growing on the surface as it can be seen after washing off of the colonies. Isolates B19a (**a**), T12a (**b**), Z17 (**c**), T67 (**d**), and B54 (**e**). **b** and **d** are transilluminated images. **f**: Pseudohyphae of a more invasive and a less invasive sector under a colony of the isolate A59 .

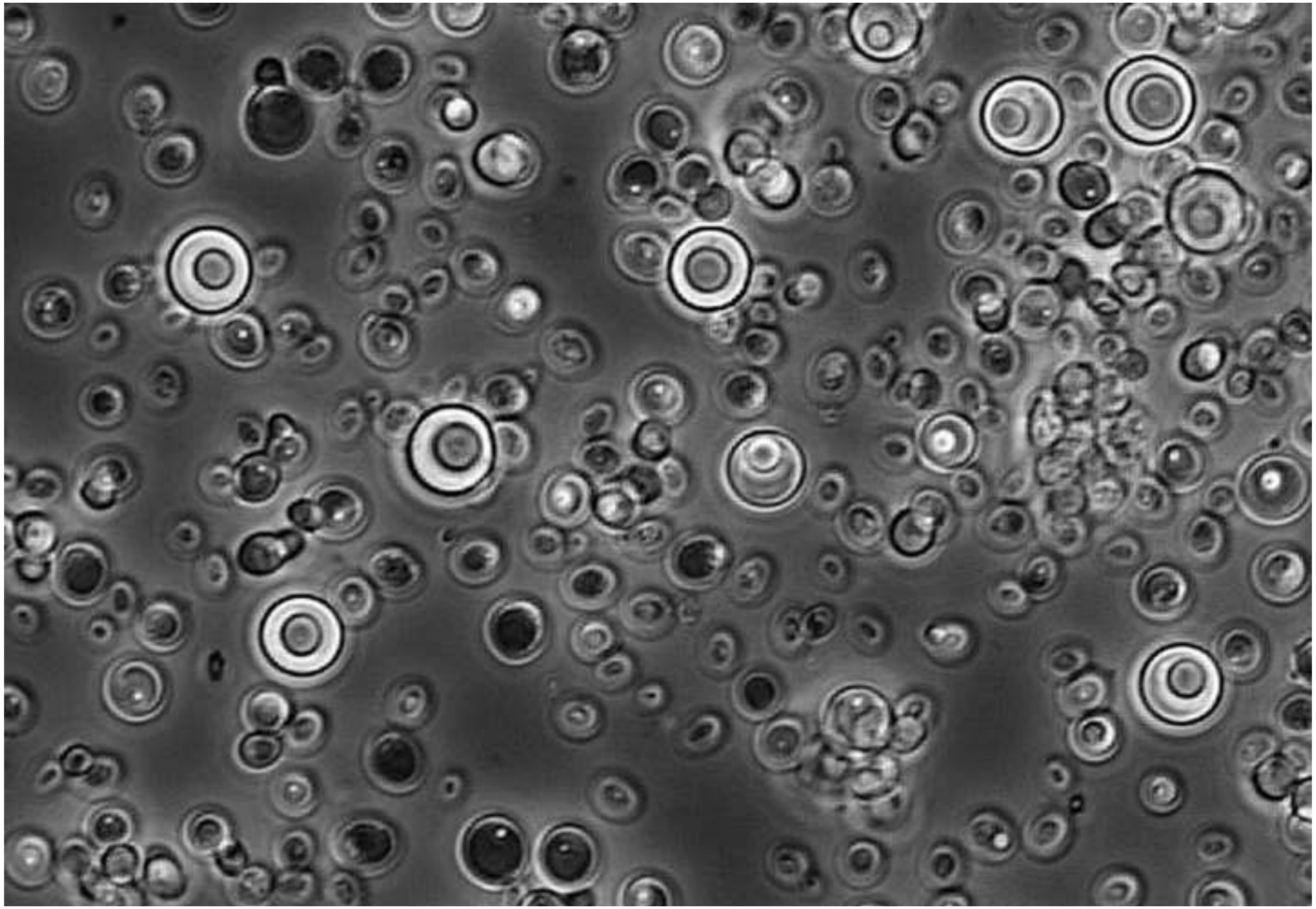

Supplementary Figure S3. Chlamidospores in a T67 culture.

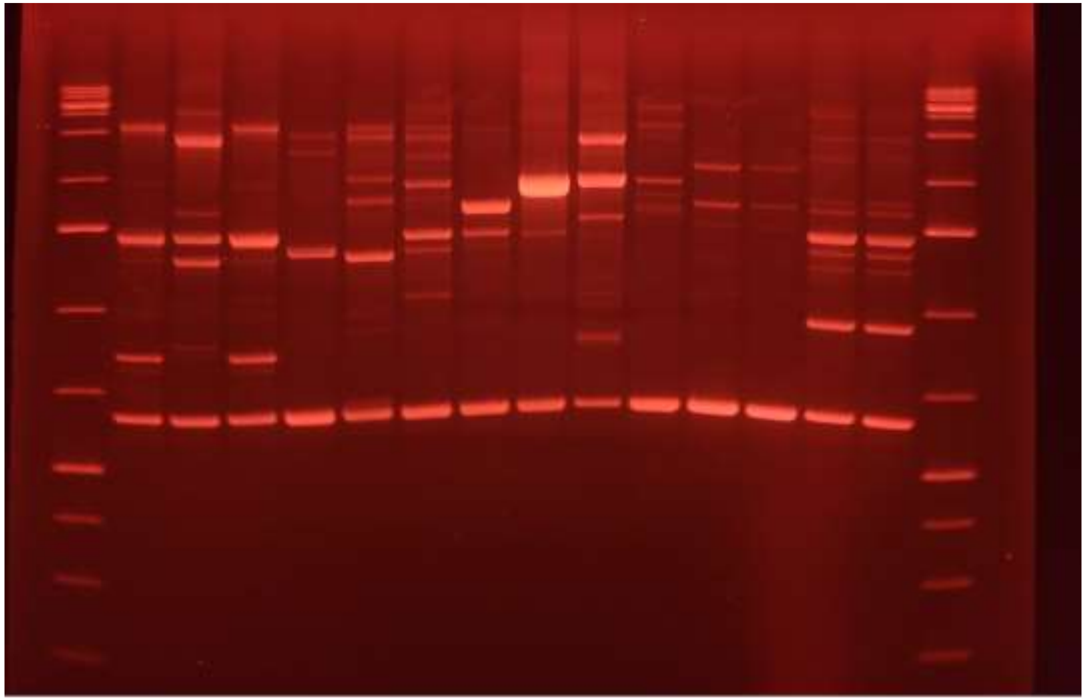

Full-length gel image for Fig. 2
